# Early Rehabilitation Index: tradução, adaptação transcultural para o português do Brasil e Early Rehabilitation Barthel Index: validação para o uso na unidade de terapia intensiva

**DOI:** 10.5935/0103-507X.20210051

**Published:** 2021

**Authors:** Nair Fritzen dos Reis, Roberta Rodolfo Mazzali Biscaro, Fernanda Cabral Xavier Sarmento Figueiredo, Elizabeth Cristiane Buss Lunardelli, Rosemeri Maurici da Silva

**Affiliations:** 1 Programa de Pós-Graduação em Ciências Médicas, Hospital Universitário Professor Polydoro Ernani de São Thiago, Universidade Federal de Santa Catarina - Florianópolis (SC), Brasil.

**Keywords:** Estado funcional, Reabilitação, Estudo de validação, Psicometria, Cuidados críticos, Unidades de terapia intensiva

## Abstract

**Objetivo:**

Traduzir, adaptar transculturalmente para o português do Brasil o instrumento *Early Rehabilitation Index* e validar para uso na unidade de terapia intensiva o instrumento *Early Rehabilitation Barthel Index*, para avaliação do estado funcional.

**Métodos:**

Foram executadas as seguintes etapas: preparação, tradução, reconciliação, tradução reversa, revisão, harmonização, pré-teste e avaliação psicométrica. Após esse processo inicial, a versão em português foi aplicada por dois avaliadores em pacientes que permaneciam pelo menos 48 horas internados na unidade de terapia intensiva. Verificou-se a confiabilidade da escala por meio da consistência interna, da confiabilidade entre avaliadores e do efeito piso e teto. Para a validade de constructo, correlacionou-se o *Early Rehabilitation Barthel Index* com instrumentos que usualmente são utilizados para avaliação do estado funcional na unidade de terapia intensiva.

**Resultados:**

Participaram 122 pacientes com mediana de idade de 56 [46,8 - 66] anos. O *Early Rehabilitation Barthel Index* teve confiabilidade adequada com coeficiente alfa de Cronbach de 0,65. A confiabilidade entre avaliadores foi excelente, com coeficiente de correlação intraclasse de 0,94 (IC95% 0,92 - 0,96) e moderado a excelente com índice de concordância de kappa de 0,54 a 1,0. Os efeitos piso e teto foram mínimos. Observou-se a validade do *Early Rehabilitation Barthel Index* por meio das correlações com o escore total do Perme Escore (rô = 0,72), da Escala de Estado Funcional em UTI (rô = 0,77), do *Physical Function in Intensive Care Test-score* (rô = 0,69), do *Medical Research Council sum score* (rô = 0,58), além das dinamometrias de preensão palmar (rô = 0,58) e manual de coxa (rô = 0,55), todos com p < 0,001.

**Conclusão:**

A versão adaptada do *Early Rehabilitation Index* para o português brasileiro e na sua totalidade, *Early Rehabilitation Barthel Index* é confiável e válida para avaliação do estado funcional dos pacientes na alta da unidade de terapia intensiva.

## INTRODUCTION

A longer time on bed restriction during hospitalization in the intensive care unit (ICU), combined with vital organ dysfunction, sepsis, hypoxemia, and neuromuscular toxicity due to medication use, impair the performance of the cardiovascular and musculoskeletal systems and thus cause a decline in functional status.^1)^

Assessing the functional status of these patients and starting an early mobilization program in the ICU can increase the success rate of weaning from mechanical ventilation (MV), shorten the ICU and hospital stays, and improve the quality of life.^2,3^ For this assessment, a reliable, reproducible, and valid instrument should be chosen.^4^

Several scales have been developed in recent years to evaluate the functional aspects of patients admitted to the ICU,^5^ and existing scales meant for other populations have also been used for this purpose.^4^ This is done to standardize the physical therapy outcome and measure the progression of critically ill patients during their ICU and hospital stays. Among these scales, an extension of the Barthel Index (BI), called the Early Rehabilitation Barthel Index (ERBI), is extensively used in Germany to assess acute neurological patients.^6^

The ERBI is the sum of the Early Rehabilitation Index (ERI) and the BI. The ERI comprises relevant items for early rehabilitation assessment of acute patients, including intensive medical monitoring, tracheostomy tube use and management, MV use, confusional state, behavioral disturbances, communication deficit, and feeding assistance. There are seven items, which if applicable have a negative value of -50 or -25 points.^7)^ The BI is a widely known scale that was created to evaluate the response to rehabilitation of individuals with chronic neurological disease. Its objective is to measure the ability to perform 10 activities of daily living (ADLs) independently, with some help or completely depending on help.^8^ The BI has already been translated and adapted to Brazilian Portuguese^9^ and validated for elderly outpatients.^10^ To calculated the ERBI score, it is necessary to add the ERI (-325 to 0 points) to the BI (0 to 100 points), which results in an ERBI total score of -325 to 100 points.^7^ The scale showed high interrater reliability and validity compared with other neurological assessment scales.^6,7^

The ERBI was developed to increase the sensitivity to changes in the BI and to monitor the progression of patients during the treatment phase,^6^ and it can be used to improve the characterization of critically ill patients. Cross-cultural adaption is the best choice for assessment instruments available in the health field because it allows them to be applied in any country, culture, and language.^11^

The objective of this study was to translate and cross-culturally adapt the ERI to Brazilian Portuguese and to evaluate the psychometric properties of the ERBI at discharge from the intensive care unit.

## METHODS

A methodological study (translation and adaptation) combined with a cross-sectional observational study (validation) was conducted in the ICU of *Hospital Universitário Professor Polydoro Ernani de São Thiago* of *Universidade Federal de Santa Catarina* from January to August 2018. The study was approved by the university’s Ethics Committee in Research on Human Beings under protocol no. 63173716.0.0000.0121. The study began with the process of translation and cross-cultural adaptation of the ERI into Brazilian Portuguese according to the recommendations of the Principles of Good Practice for the Translation and Cultural Adaptation Process for Patient-Reported Outcomes,^12^ followed by the evaluation of the psychometric properties of the ERBI through the COnsensus-based Standards for the selection of health Measurement INstruments (COSMIN).^13^

### Translation and cross-cultural adaptation process

The process of translation and cross-cultural adaptation of the ERI into Brazilian Portuguese had the following steps: preparation, which consisted of obtaining the authorization of Dr. Jens D. Rollnik, Institute for Neurorehabilitation Research (InFo), Germany, to use the instrument; translation from English to Portuguese, performed independently by two translators native in Portuguese and fluent in English, one familiar and the other unfamiliar with the scale (T1 and T2); reconciliation, in which the two translated versions were compared and analyzed by a coordinator (any existing discrepancies were analyzed and discussed, and this process resulted in a version translated by consensus - T12); back-translation into English, in which the Brazilian Portuguese version was translated back into English by two other independent translators, native in English and fluent in Portuguese (none of them had contact with the original English versions - BT1 and BT2); revision and harmonization of the back-translations, in which a committee composed of three researchers reviewed the back-translations of the scales against the original version, in search of possible discrepancies and in order to make the necessary adjustments, and a final version of the back-translation was created and sent to the original author of the scale for approval and comments; lastly, a final version in Portuguese was created by the committee (BT12), called the *Índice de Reabilitação Precoce* (IRP), and the pretest was performed: with the final version in Portuguese, a pilot study was conducted in which two physical therapists applied the scale to volunteers. The objective of this phase was to identify interpretation problems and difficulties in applying the scale (Table 1S - Supplementary material).

### Validation process

After final approval of the translation and cross-cultural adaptation process, the new version needed to demonstrate adequate measurement properties.^14^ The relevant guidelines say that eight main attributes should be evaluated to confirm the adequacy of the instrument: content, criterion, and construct validity; internal consistency; reproducibility; responsiveness; floor and ceiling effects; and interpretability.^15^ Hereafter, we will refer to the ERBI instrument in its entirety as *Índice de Reabilitação Precoce e Barthel* (IRPB).

For this study, the psychometric properties of the IRPB at discharge from the ICU were evaluated through the following attributes: internal consistency, which is the measure of whether the scale items correlate with each other;^15^ interrater reliability, which refers to the degree to which the measurement is free of measurement error when measured by different individuals at the same time;^15,16^ interrater measurement error, which consists of systematic and random errors of a patient’s score and how close the scores are in repeated measures.^15,16^ The measurement error is expressed by the standard error of measurement (SEM).^15^ Floor and ceiling effects are present when more than 15% of participants achieve the highest and lowest scores, respectively, on the scale.^15^ Construct validity refers to the degree to which the scores of the instrument are related to other measures that measure the same concept through hypotheses.^15^

### Participants

All patients who were consecutively admitted to the general ICU and were aged ≥ 18 years were eligible for the study. The inclusion criterion adopted was completing 48 hours of ICU stay and signing of the informed consent form by a family member, a guardian, or the participant. Individuals who progressed to palliative care, brain death, or death, those who were transferred to another hospital during their ICU stay, those unable to perform at least three of the five essential commands (open and close their eyes, raise their eyebrow, stick out their tongue, move their head, and look at the evaluator),^17^ upper- or lower-limb amputees, and those who withdrew by the decision of their relative, their guardian, or themselves were excluded.

### Study variables and data collection

Based on the fulfillment of the inclusion criteria, data including age, sex, body mass index (BMI), Acute Physiology and Chronic Health Evaluation II (APACHE II) score, Simplified Acute Physiology Score III (SAPS III) score, Charlson comorbidity index score, reason for ICU admission, lengths of ICU and hospital stays, use and duration of invasive MV (IMV), and hospital outcome were collected.

Patients were followed up until discharge from the ICU. The assessments were performed at ICU discharge or within 24 hours after discharge by two physical therapists with the same level of clinical experience. This assessment consisted of the application of the following instruments (Table 2S - Supplementary material): ERI/IRP, which measures seven items relevant to early rehabilitation assessment in acute patients, with a total score ranging from -325 to 0 points;^6^ BI, which measures the ability to perform 10 ADLs, with a total score ranging from 0 to 100 points;^8,10)^ Perme ICU Mobility Score (Perme Score), which measures the mobility of ICU patients, with a total score ranging from 0 to 32 points;^18,19^ Functional Status Score for the ICU (FSS-ICU), which measures five functional tasks with a total score ranging from 0 to 35 points;^20,21^ Physical Function in Intensive Care Test-scored (PFIT-s), which measures the patient’s ability to perform four tasks, with a total score ranging from 0 to 12 points (ordinal scale);^22^ Medical Research Council sum score (MRC-SS), which evaluates peripheral muscle strength, with a total score ranging from 0 to 60 points;^23^ handgrip dynamometry to measure handgrip strength in the dominant hand, using a Jamar dynamometer (Jamar Plus+, model 12-0604, Bolingbrook, Illinois, United States) and following the recommendations of the American Society of Hand Therapists (ASHT);^24^ and hand-held dynamometry for isometric strength, by using a dynamometer to measure the knee extensor strength (Lafayette instrument, model 01165, Lafayette, Indiana, United States).^25^ The measurement of muscle strength by dynamometry was performed three times, and the one with the highest score was used for evaluation.^24^ For all instruments, a high score reflects better functional status or muscle strength.

Some instruments used in this study have items that are evaluated in the same way (for example, transfer from sitting to standing). As a result, an evaluation sequence and form were created to prevent the patient from performing the same task more than once and experiencing fatigue and to allow the scoring of several scales at the same time. The assessment was conducted by a principal evaluator, who applied the tests and scales, and by a secondary evaluator, who only observed the procedure. Both were trained and familiarized with the evaluation sequence. The roles of the principal evaluator and observer switched at each evaluation. To avoid bias, after the evaluation, the two evaluators filled out the scoring sheet of the instruments without contact or discussion between them. The same evaluation order was used for all patients and lasted approximately 1 hour.

For better understanding, the Perme score, FSS-ICU, and PFIT-s will be referred to as “functional scales specific to the ICU” and the MRC-SS and the handgrip and knee extensor dynamometry as “muscle strength measurements”.

### Statistical analysis

Measures of central tendency and dispersion, including arithmetic mean, standard deviation (SD), median, interquartile range (IQR_25-75%_), frequency, and percentage, were applied to the variables according to the normality and type of the data. The normality of the data was assessed using the Kolmogorov-Smirnov test.

To determine the internal consistency, Cronbach’s alpha coefficient was calculated for the scale in its entirety and after excluding each item one by one. The gold-standard minimum alpha value of 0.70 was adopted.^15^

The interrater reliability was assessed using the two-way random intraclass correlation coefficient (ICC), which measures absolute agreement, for single measures and for the total score, and the kappa agreement index for each item individually (1 to 17). A value above 0.70 is recommended as a minimum standard for reliability,^15^ and values above 0.75 were considered excellent.^26^ For the analysis of interrater measurement error, the SEM of agreement (SEM_agreement_) and the minimum detectable change in the individual (MDC_individual_) were calculated.

The floor and ceiling effects were determined from the proportions of the assessments that obtained the lowest and highest scores. The effects are considered present when more than 15% of the respondents achieve the lowest and highest possible scores. This indicates that the instrument has a limitation in its content validation and the subjects cannot be distinguished from each other, which would reduce its reliability.^15^

Although several instruments have been developed in recent years, none has yet been considered the gold standard for assessing the functional status of critically ill patients. Thus, construct validation was performed by correlating the IRPB with functional scales specific for the ICU and muscle strength measurements. The hypothesis that the IRPB score would have a positive correlation with the other measures was adopted, with an r of at least 0.75.^15^ The Spearman correlation coefficient was used for the analyses.^27^

A sample size of at least 100 participants is recommended and is considered excellent,^28^ which was the objective of this study. All patients included were considered for the analyses of reliability, floor and ceiling effects, and construct validity. There were no missing data, and when only one IRPB value was needed, the values from the principal evaluator were considered. All analyses were performed using the Statistical Package for the Social Sciences (SPSS), version 22.0 (SPSS Inc., Chicago, Illinois, United States). For all analyses, the significance level adopted was 5%.

## RESULTS

**Figure 1:**
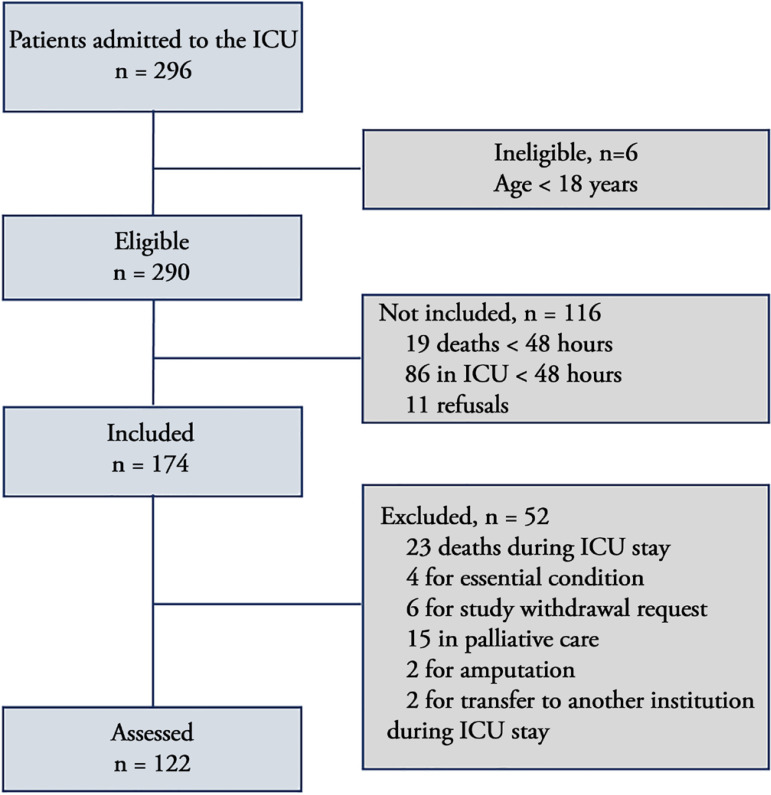
Eligibility and inclusion criteria ICU - intensive care unit.

From January to August 2018, 290 patients admitted to the ICU were eligible. After exclusion, 122 patients were included in the study (Figure 1). Their baseline and clinical characteristics are shown in table 1. The values for the functional scales and muscle strength measurements applied at ICU discharge are shown in table 2.

**Table 1:** Baseline and clinical characteristics of the sample

Characteristics	
Age (years)	56 [46.8 - 66]
Male	62 (51)
BMI (kg/m²)	25 [21.1 - 29.2]
Low weight (< 18.5kg/m²)	9 (7)
Adequate weight (≥ 18.5 and < 25kg/m²)	51 (42)
Overweight (≥ 25 and < 30kg/m²)	36 (30)
Obesity (≥ 30kg/m²)	26 (21)
SAPS III	60.2 ± 14.6
APACHE II	20.9 ± 8.2
Charlson Comorbidity Index	3 [1-4]
Reason for ICU admission	
Sepsis	28 (23)
Postoperative period of elective surgery	23 (19)
Primary cardiovascular disorder	17 (14)
Primary central nervous system disorder	15 (12)
Postoperative period of emergency surgery	14 (11)
Primary respiratory disorder	10 (8)
Primary digestive system disorder	9 (7)
Other	6 (5)
Use of IMV	83 (68)
IMV (days)	5 [3 - 8]
Length of ICU stay (days)	7 [5 - 11]
Length of hospital stay (days)	22 [14 - 30.3]
Hospital outcome	
Hospital discharge	107 (88)
Death	13 (11)
Transfer to another institution	2 (2)

BMI - body mass index; SAPS III - Simplified Acute Physiology Score III; APACHE II - Acute Physiology and Chronic Health Evaluation; ICU - intensive care unit; IMV - invasive mechanical ventilation. Results are median [interquartile range], n (%), or mean ± standard deviation.

**Table 2:** Functional status and muscle strength characteristics at discharge from the intensive care unit

Variable	
*Índice de Reabilitação Precoce*	
Primary evaluator	-50 [-50 - 0]
Secondary evaluator	-50 [-50 - 0]
Barthel Index	
Primary evaluator	25 [10 - 60]
Secondary evaluator	25 [15 - 55]
*Índice de Reabilitação Precoce e Barthel*	
Primary evaluator	-20 [-46.3 - 35]
Secondary evaluator	-15 [-45 - 40]
Perme Score	25.5 [15 - 30]
FSS-ICU	23 [11 - 31.3]
PFIT-s	8 [5 - 10]
MRC-SS	56 [50.8 - 59]
Muscle weakness (< 48 points)	21 (17)
Handgrip dynamometry	16.5 ± 9.4
Muscle weakness (women < 7kgf and men < 11kgf)	24 (20)
Hand-held knee extensor dynamometry	7.8 ± 3.5

FSS-ICU - Functional Status Score for the ICU; PFIT-s - Physical Function in Intensive Care Test score; MRC-SS - Medical Research Council sum score. Results are median [interquartile range], n (%), or mean ± standard deviation.

### Translation and cross-cultural adaptation

During the development of the Portuguese version (IRP), the translated versions were similar. In the back-translation to English, no differences were found from the original scale that changed the meaning of any item.

In the pretest phase, the physical therapists reported uncertainty regarding the item “swallowing disorders in need of supervision” given two situations: whether the option should be checked only if the swallowing disorder was diagnosed by the speech therapist and whether the use of a feeding tube should be considered help. The question raised was answered by Dr. Rollnik, who explained that the scale also evaluated the need for help and supervision by the team for feeding. Thus, in consensus among the evaluators, it was defined that the item should be checked when the patient was using a feeding tube (because the patient needed a change of diet) and when the patient needed help to eat orally. As a matter of routine in Brazilian ICUs, the item was replaced by “help and/or supervision in feeding”, and its interrater agreement was excellent (kappa of 0.88; p < 0.001). The translated scale is included as table 3.

**Table 3:** *Índice de Reabilitação Precoce*, version translated to Brazilian Portuguese

Item	Valor	
	Sim	Não
*1. Monitorização de cuidados intensivos*	- 50	0
*2. Supervisão e cuidados com traqueostomia*	- 50	0
*3. Ventilação mecânica intermitente ou contínua*	- 50	0
*4. Estado de confusão com necessidade supervisão*	- 50	0
*5. Distúrbios de comportamento com necessidade de cuidados especiais (paciente representa risco para ele mesmo ou para seu ambiente)*	- 50	0
*6. Défice grave de comunicação*	- 25	0
*7. Assistência e/ou supervisão na alimentação*	- 50	0
*Total (IRP)*	- 325 a 0 pontos	
*+ IB*	0 a + 100 pontos	
*Total do IRPB (IRP + IB)*	- 325 a - 100 pontos	

*IRP - Índice de Reabilitação Precoce; IB - Índice de Barthel; IRPB - Índice de Reabilitação Precoce e Barthel.*

### Reliability and measurement error

The IRPB showed internal consistency, with a Cronbach’s alpha of 0.65, a value close to that desired by the study. The interrater reliability, based on the total value of the scale, was considered excellent, and when the items were evaluated individually, the values ranged from moderate to excellent. The SEM_agreement_ was 31.58, and the MDC_individual_ was 87.54 points. There was no floor effect of the IRPB, and the ceiling effect value found was below the 15% limit (Table 4).

**Table 4:** Internal consistency and interrater reliability based on the total and per-item score (1 to 17) of the *Índice de Reabilitação Precoce e Barthel*

IRPB	Cronbach's α	ICC (95%CI)	Floor effect n (%)	Ceiling effect n (%)
**Total score**	0.65	0.94 (0.92 - 0.96)	0 (0)	1 (0.8)
**Items**	**Cronbach's ** **α** ** if the item is excluded**	**k**	**p value**	
1. Monitoring	0.68	1.0	< 0.001	
2. Use of TCT	0.62	1.0	< 0.001	
3. Use of MV	0.64	0.85	< 0.001	
4. Confusional state	0.63	0.68	< 0.001	
5. Behavioral disturbance	0.63	0.55	< 0.001	
6. Communication deficit	0.63	0.79	< 0.001	
7. Feeding assistance	0.60	0.88	< 0.001	
8. Feeding	0.61	0.82	< 0.001	
9. Grooming	0.64	0.54	< 0.001	
10. Toilet use	0.64	0.83	< 0.001	
11. Bathing	0.64	0.65	< 0.001	
12. Bowels control	0.64	0.68	< 0.001	
13. Bladder control	0.63	0.81	< 0.001	
14. Dressing	0.64	0.89	< 0.001	
15. Bed-to-chair transfer	0.60	0.88	< 0.001	
16. Stairs	0.62	0.92	< 0.001	
17. Mobility	0.60	0.94	< 0.001	

IRPB - *Índice de Reabilitação Precoce e Barthel*; ICC - intraclass correlation coefficient; 95%CI - 95% confidence interval; TCT - tracheostomy; MV - mechanical ventilation.

### Construct validity

In the correlation of the IRPB with the Perme score and FSS-ICU, the study hypothesis was accepted, as a strong positive correlation was found. In contrast, the muscle strength measurements and PFIT-s showed a moderate correlation (Table 5).

**Table 5:** Correlations between the *Índice de Reabilitação Precoce e Barthel* and functional scales specific to the intensive care unit and muscle strength tests

	IRPB	
	ρ	p value
Perme score	0.72	< 0.001
FSS-ICU	0.77	< 0.001
PFIT-s	0.69	< 0.001
MRC-SS	0.58	< 0.001
Handgrip dynamometry	0.58	< 0.001
Hand-held knee extensor dynamometry	0.55	< 0.001

IRPB - *Índice de Reabilitação Precoce e Barthel;* FSS-ICU - Functional Status Score for the ICU; PFIT-s - Physical Function in Intensive Care Test score; MRC-SS - Medical Research Council sum score.

## DISCUSSION

The IRP, translated and adapted to Brazilian Portuguese, was found to be easy to understand. The version in its entirety, the IRPB, was reliable when applied by different evaluators to critically ill patients at the time of ICU discharge.

The internal consistency of the IRPB for use at ICU discharge was lower than the adopted gold standard, which could indicate a low correlation between the items.^15^ Some aspects are believed to have influenced this result and should be taken into consideration. First, the scale was originally developed for the assessment of acute neurological patients, without any internal consistency value reported,^6^ and in this study, the IRPB was used in a population with different diagnoses, predominantly sepsis and recovery from elective surgery. The application of a scale in different populations can cause variations in its internal consistency.^29^ In addition, the IRPB is a sum of two indices that measure different aspects, as a way to better characterize the patient’s rehabilitation (for example, monitoring, MV use, confusional state, behavior, and ADLs), which could influence the correlation between its items individually but which does not invalidate its importance and applicability. Thus, the internal consistency of the IRPB reflects an acceptable correlation for its application in a general ICU population at the time of discharge.^29,30^

The best internal consistency value was achieved when the item “intensive care supervision” was removed. This could be because the patients were in an intensive care environment and under constant supervision; the interrater agreement for this item was perfect. However, we think it is helpful to keep this item in this tool for assessing the progression of the functional status and care of the patient throughout hospitalization and after discharge.

When the interrater reliability of the items was individually evaluated by the kappa agreement index, those with lower values were “mental confusion” and “behavioral disturbance”. In the original study of the scale, similar values were found through correlation analysis.^6^ These lower values may have occurred due to the inherent subjectivity of these items and the dependence on the evaluators’ interpretation. Nevertheless, the variation in agreement was moderate to excellent, which can be considered acceptable. However, to enable greater accuracy in the scoring of these items, a tool that objectively diagnoses delirium in critically ill patients could be applied, such as the Confusion Assessment Method for Intensive Care Unit.^31^

The interrater reliability of the total score of the scale, as judged by the ICC, was also considered excellent. These results, combined with floor and ceiling effects of less than 15%, suggest that the IRPB is adequate and reliable for application at ICU discharge. In addition, according to the COSMIN classification, the interrater measurement error was considered indeterminate^32^ because there is still no minimum change value deemed important for the IPRB, which will be necessary for its complete analysis. However, due to the variation in the IRPB score (-325 to 100 points), the SEM_agreement_ and the MDC_individual_ are considered acceptable.

The IRPB was shown to have construct validity when correlated with other instruments that assess the functional status and muscle strength of patients in the ICU. The Brazilian version correlated strongly and positively with the Perme score and FSS-ICU. These scales were created specifically for the ICU and evaluate the functional status predominantly through the amount of help required for transfers and the barriers to mobilization. These results may be explained by the fact that the IRPB has some similar items, focusing on the care and supervision that the patient requires, in addition to some mobility aspects originating from the BI. With the PFIT-s, MRC-SS, and handgrip and knee extensor dynamometry, the correlations were positive and moderate. The PFIT-s is a score that has four items, two of which refer to the muscle strength domain.^33^ Most likely this was the reason the correlations between the IRPB and these instruments did not come out as we hypothesized.

Some limitations of this study should be considered. The assessments were performed preferably in the ICU or with a maximum tolerance of up to 24 hours after discharge from the ICU. To minimize the possible effects of changing the assessment site, some items (e.g., using the bathroom, bathing, bowel and bladder continence) were scored according to the reports of the patients and/or the multidisciplinary team specifically for the reference site, the ICU. The IRPB should be used with caution in future studies and in clinical practice, and it should be remembered that the present study is single-center, with patients admitted to a general ICU with different diagnoses, baseline characteristics, and ventilation statuses. Additional studies could test other psychometric properties that were not considered in this study and/or other populations.

The IRPB assesses critically ill patients through different aspects: need for monitoring, use of MV and tracheostoma, confusional state and behavior, communication, feeding, ADLs, and mobility. Learning more about these components would allow for a better differentiation of how these patients leave the ICU and progress during rehabilitation. This version translated and cross-culturally adapted for Brazil allows access by professionals to the tool and a similar description of the disease or treatment for comparison with studies from other countries,^11^ which can ensure a better quality of patient care and research.^18^

## CONCLUSION

The version of the Early Rehabilitation Index adapted for Brazilian Portuguese, the *Índice de Reabilitação Precoce*, was easy to understand and apply. The Early Rehabilitation Barthel Index, or *Índice de Reabilitação Precoce e Barthel*, is sufficiently reliable and can be applied by different evaluators, in addition to having satisfactory construct validity, and is a tool that can be used to assess functional status at the time of discharge from the intensive care unit.
