# Supplementary material for: Early Rehabilitation Index: tradução, adaptação transcultural para o português do Brasil e Early Rehabilitation Barthel Index: validação para o uso na unidade de terapia intensiva
Source: Rev Bras Ter Intensiva. 2021 Jul-Sep;33(3):353–61. doi: 10.5935/0103-507X.20210051 (PMC8555403; doi:10.5935/0103-507X.20210051)
Supplement: Supplementary file 1 [file rbti-33-03-0353-suppl01.pdf]

Early Rehabilitation Index: translation and cross-cultural adaptation to Brazilian Portuguese; and Early Rehabilitation Barthel Index: validation for use in the intensive care unit

Early Rehabilitation Index: tradução, adaptação transcultural para o português do Brasil e Early Rehabilitation Barthel Index: validação para o uso na unidade de terapia intensiva

Nair Fritzen dos Reis[1], Roberta Rodolfo Mazzali Biscaro[1], Fernanda Cabral Xavier Sarmento Figueiredo[1], Elizabeth Cristiane Buss Lunardelli[1], Rosemeri Maurici da Silva[1]

Table 1S - Translation and back-translation process of the Early Rehabilitation Index

Original version	T1	T2	T12	BT1	BT2	BT12
1. Intensive medical monitoring	*Monitorização intensiva*	*Monitorização de cuidados intensivos*	*Monitorização de cuidados intensivos*	Intensive care monitoring	Intensive care monitoring	Intensive care monitoring
2. Tracheostoma requiring special treatment (suctioning)	*Cuidados e supervisão da traqueostomia*	*Supervisão e cuidados com a traqueostomia*	*Supervisão e cuidados com traqueostomia*	Supervision and tracheostomy	Tracheostomy supervision and care	Tracheostomy supervision and care
3. Intermittent (or continuous) mechanical ventilation	*Ventilação mecânica contínua ou intermitente*	*Ventilação mecânica intermitente ou contínua*	*Ventilação mecânica intermitente ou contínua*	Intermittent or continuous mechanical ventilator support	Intermittent or continuous mechanical ventilation	Intermittent or continuous mechanical ventilation
4. Confusional state requiring special supervision	*Paciente confuso (com necessidade de supervisão)*	*Estado de confusão com necessidade de supervisão*	*Estado de confusão com necessidade de supervisão*	Altered mental status requiring constant supervision	State of confusion with need for supervision	State of confusion with need for supervision
5. Behavioral disturbances requiring special care (patient poses a risk to himself or his environment)	*Distúrbios de comportamento (paciente apresenta perigo para si mesmo ou outro)*	*Distúrbios de comportamento com necessidade de cuidados especiais (paciente apresenta risco para ele mesmo ou para seu ambiente)*	*Distúrbios de comportamento com necessidade de cuidados especiais (paciente apresenta risco para ele mesmo ou para seu ambiente)*	Behavioral changes with need for supervision (patient at risk to harm self or others)	Behavioral disorders necessitating special care (the patient is a risk to himself or to his environment)	Behavioral disorders requiring special care (the patient is a risk to himself or to his environment)
6. Severe communication deficits	*Prejuízo grave de comunicação*	*Deficits graves de comunicação*	*Deficit grave de comunicação*	Severe communication impairment	Severe lack of communication	Severe communication impairment
7. Swallowing disorders requiring special supervision	*Paciente disfágico com necessidade de supervisão*	*Distúrbios de deglutição com necessidade de supervisão*	*Distúrbios de deglutição com necessidade de supervisão*	Swallowing difficulty requiring constant supervision	Deglutition disorders requiring supervision	Swallowing disorders requiring supervision

T1 - translator 1; T2 - translator 2; T12 - synthesis of translations T1 and T2; BT1 - back: translation 1; BT2 - back: translation 2; BT12 - synthesis of back: translations BT1 and BT2.

Table 2S - Description of the functional scales and peripheral muscle strength scale

Scale	Objective	Score	Items evaluated	Where to find
ERI/IRP	Evaluate the early rehabilitation of acute patients	–325 to 0 points	Monitoring, use of tracheostomy, use of MV, confusional state, behavioral disturbance, communication deficit, feeding assistance	DOI: 10.1055/s-0031-1273728
BI	Evaluate the rehabilitation of patients with chronic neurological disease	0 to 100 points	Feeding: 0 = unable, 5 = needs help or 10 = independent;<br>Grooming: 0 = needs help or 5 = independent;<br>Toilet use: 0 = dependent, 5 = needs some help or 10 = independent;<br>Bathing: 0 = dependent or 5 = independent;<br>Bowels: 0 = incontinent, 5 = occasional accident or 10 = continent;<br>Bladder: 0 = incontinent, 5 = occasional accident or 10 = continent;<br>Dressing: 0 = dependent, 5 = some help or 10 = independent;<br>Bed-to-chair transfer: 0 = unable, 5 = major help, 10 = minor help or 15 = independent;<br>Stairs: 0 = unable, 5 = needs help (verbal or physical) and 10 = independent;<br>Mobility: 0 = immobile or ≤ 50 m, 5 = wheelchair independent, 10 = walks > 50 m with help of one person; 15 = independent > 50 m alone with or without the use of a cane	DOI: 10.1590/S0103-21002010000200011<br>The authors provide a detailed description of what to consider when scoring each item.
Perme Score	Measure patient mobility in the ICU	0 to 32 points	Mental status, potential mobility barriers, functional strength, bed mobility, transfers, gait, endurance	DOI: 10.1590/s1806-37562015000000301<br>DOI: 10.1590/s1806-37562015000000301<br>Ask the authors to provide the supplementary file with the Perme score.
FSS-ICU	Evaluate physical function by performing 5 functional tasks	0 to 35 points	Rolling, transfer from spine to sit, sitting at the edge of bed, transfer from sit to stand, walking	DOI: 10.5935/0103-507X.20170006<br>Request from the authors the FSS-ICU file or access the website www.ImproveLTO.com-Section "Instruments" – "Physical Function" – "Tests" – "Functional Status Score for the Intensive Care Unit (FSS-ICU) – Other languages" – Portuguese version.
PFIT-s	Evaluate physical function by performing 4 tasks	Ordinal: 0 to 12 points<br>Interval: 0 to 10 points	Amount of assistance for sit-to-stand transfers, marching in place, knee extensor and flexor muscle strength	DOI: 10.36416/1806-3756/e20180366<br>Access the supplementary material provided in the article.
MRC-SS	Measure the peripheral muscle strength of 6 muscle groups bilaterally	0 to 60 points	Movements: shoulder abduction, elbow flexion, wrist extension, hip flexion, knee extension, ankle dorsiflexion	DOI: 10.3791/2632<br>Explanation of the test with video and photos.

ERI - Early Rehabilitation Index; IRP - *Índice de Reabilitação Precoce*; MV -: mechanical ventilation; BI - Barthel Index; FSS-ICU - Functional Status Score for the ICU; ICU - intensive care unit; PFIT-s - Physical Function in Intensive Care Test score; MRC-SS - Medical Research Council sum score.
